# Supplementary material for: Autologous hGMSC-Derived iPS: A New Proposal for Tissue Regeneration
Source: Int J Mol Sci. 2024 Aug 23;25(17):9169. doi: 10.3390/ijms25179169 (PMC11395260; doi:10.3390/ijms25179169)
Supplement: Supplementary file 1 [file ijms-25-09169-s001.zip › Table S1.pdf]

hsa-let-7e	hsa-miR-34a	hsa-miR-192	hsa-miR-365
hsa-let-7g	hsa-miR-92a	hsa-miR-193a-3p	hsa-miR-374
hsa-miR-16	mmu-miR-93	hsa-miR-193b	mmu-miR-374-5p
hsa-miR-17	hsa-miR-99b	hsa-miR-195	hsa-miR-376a
hsa-miR-18a	hsa-miR-106a	hsa-miR-197	mmu-miR-379
hsa-miR-19a	hsa-miR-106b	hsa-miR-218	hsa-miR-410
hsa-miR-19b	hsa-miR-125b	hsa-miR-221	hsa-miR-411
hsa-miR-20a	hsa-miR-126	hsa-miR-224	hsa-miR-433
hsa-miR-17	hsa-miR-127	hsa-miR-296	hsa-miR-484
hsa-miR-18a	hsa-miR-128a	hsa-miR-302a	hsa-miR-487b
hsa-miR-21	hsa-miR-130a	hsa-miR-302b	hsa-miR-494
hsa-miR-23a	hsa-miR-132	hsa-miR-323-3p	mmu-miR-495
hsa-miR-24	hsa-miR-133a	hsa-miR-324-5p	hsa-miR-518d
hsa-miR-26a	mmu-miR-134	hsa-miR-328	hsa-miR-532-3p
hsa-miR-26b	mmu-miR-137	hsa-miR-331	hsa-miR-532
hsa-miR-28-3p	mmu-miR-140	hsa-miR-335	hsa-miR-574-3p
hsa-miR-29a	hsa-miR-146a	hsa-miR-339-3p	hsa-miR-597
hsa-miR-29c	hsa-miR-146b	hsa-miR-155	hsa-miR-660
hsa-miR-30b	hsa-miR-149	hsa-miR-342-3p	hsa-miR-886-5p
hsa-miR-30c	hsa-miR-150	hsa-miR-345	hsa-miR-376c

Table S1 A. List of characterized microRNAs in common in the hGMSCs and hGMSCs-derived iPS EVs.

hsa-miR-9	hsa-miR-200a	hsa-miR-431	hsa-miR-886-3p
hsa-miR-10a	hsa-miR-200b	hsa-miR-455-3p	hsa-miR-211
hsa-miR-15b	hsa-miR-202	hsa-miR-489	hsa-miR-520b
hsa-miR-18b	hsa-miR-203	hsa-miR-500	hsa-miR-520e
hsa-miR-20b	hsa-miR-204	hsa-miR-505	
hsa-miR-28	hsa-miR-205	hsa-miR-512-3p	
hsa-miR-29b	hsa-miR-210	hsa-miR-515-5p	
hsa-miR-34c	hsa-miR-215	hsa-miR-517a	
hsa-miR-95	hsa-miR-299-5p	hsa-miR-517c	
hsa-miR-103	hsa-miR-301b	hsa-miR-518e	
hsa-miR-105	hsa-miR-302c	hsa-miR-518f	
mmu-miR-124a	hsa-miR-324-3p	hsa-miR-519a	
hsa-miR-125a-5p	hsa-miR-330	hsa-miR-519d	
mmu-miR-129-3p	hsa-miR-339-5p	hsa-miR-520a	
hsa-miR-130b	hsa-miR-340	hsa-miR-520g	
hsa-miR-135a	hsa-miR-363	hsa-miR-522	
hsa-miR-135b	hsa-miR-367	hsa-miR-525-3p	
hsa-miR-148a	hsa-miR-372	hsa-miR-590-5p	
hsa-miR-181a	hsa-miR-375	hsa-miR-598	
mmu-miR-187	hsa-miR-429	hsa-miR-651	

Table S1 B. List of characterized microRNAs solely in the parental EVs group.

hsa-miR-122	hsa-miR-152
hsa-miR-138	hsa-let-7b
hsa-miR-142-3p	mmu-miR-451
hsa-miR-143	hsa-miR-486

Table S1 C. List of characterized microRNAs solely in the reprogrammed EVs group.

hsa-miR-302b	hsa-miR-127	hsa-miR-155	hsa-let-7g
hsa-miR-19b	hsa-miR-376a	hsa-miR-376c	hsa-miR-149
hsa-miR-26b	hsa-miR-484	hsa-miR-224	hsa-miR-487b
hsa-miR-26a	hsa-miR-339-3p	hsa-miR-126	hsa-miR-342-3p
mmu-miR-495	hsa-miR-532-3p	hsa-miR-197	hsa-miR-221
hsa-miR-150	hsa-miR-433	hsa-miR-30b	hsa-miR-146b
hsa-miR-597	hsa-miR-24	hsa-miR-99b	hsa-miR-411
hsa-miR-302a	hsa-miR-365	hsa-let-7e	hsa-miR-16
hsa-miR-218	hsa-miR-574-3p	hsa-miR-28-3p	
hsa-miR-328	hsa-miR-532	hsa-miR-133a	
hsa-miR-494	hsa-miR-23a	hsa-miR-886-5p	

Table S1 D. List of significantly expressed microRNAs in the EVs of the reprogrammedversus the parental ones analyzed.
