# Supplementary figures and images for: Autologous hGMSC-Derived iPS: A New Proposal for Tissue Regeneration
Source: Int J Mol Sci. 2024 Aug 23;25(17):9169. doi: 10.3390/ijms25179169 (PMC11395260; doi:10.3390/ijms25179169)

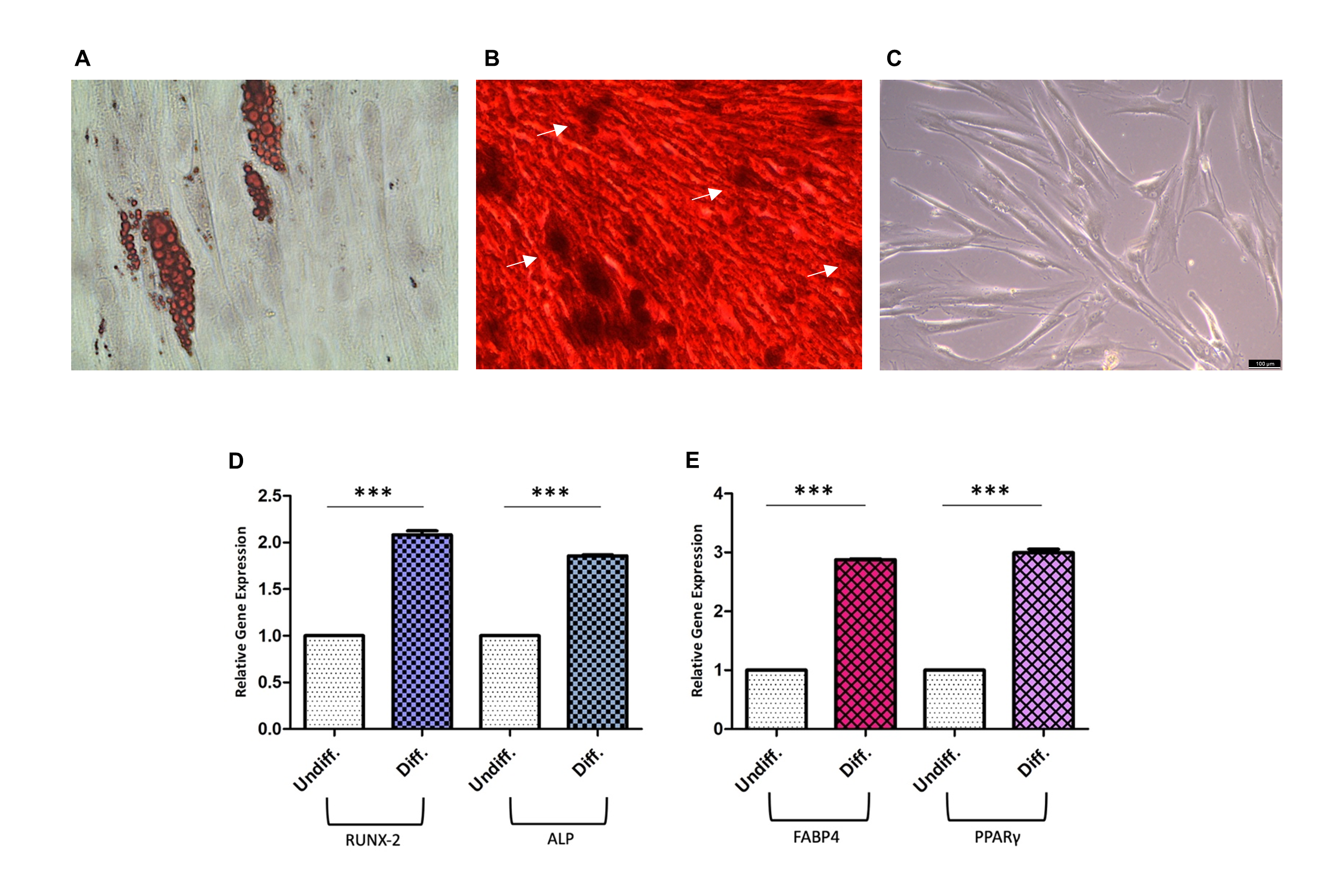

Supplement: Supplementary file 1 [file ijms-25-09169-s001.zip › Figure S1.tif]

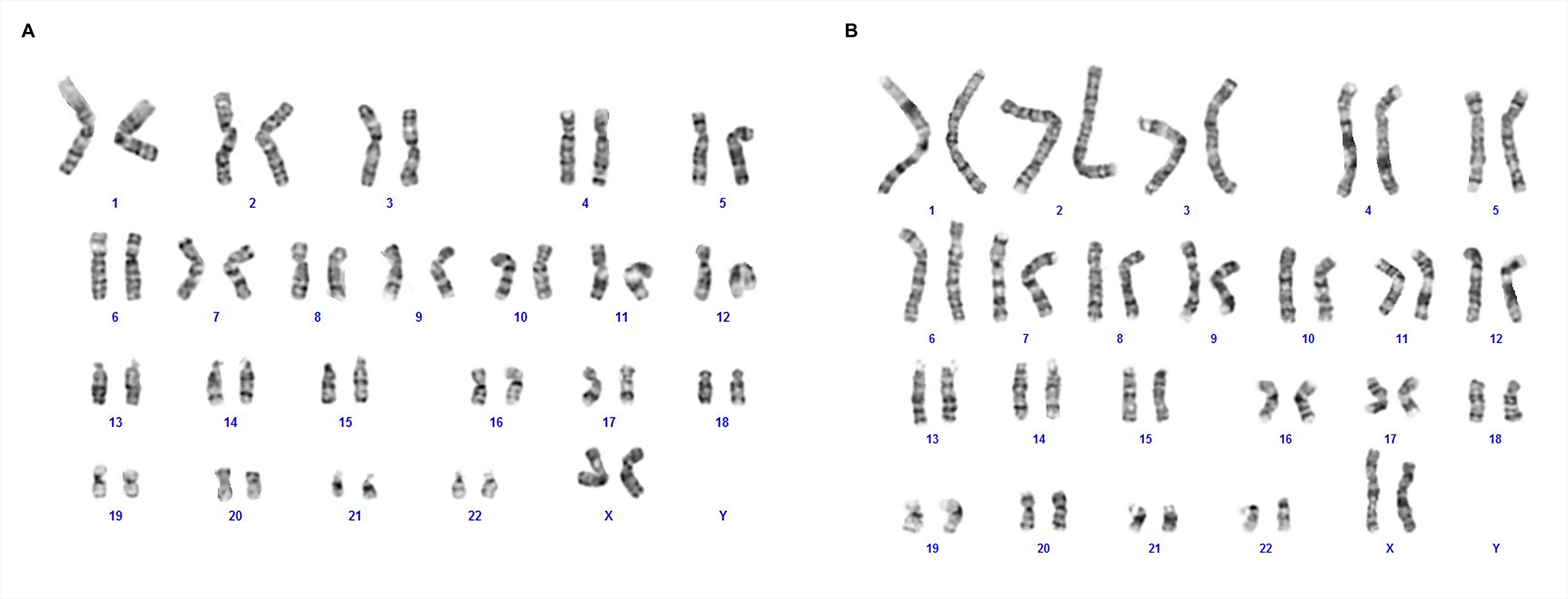

Supplement: Supplementary file 1 [file ijms-25-09169-s001.zip › Figure S2.tif]

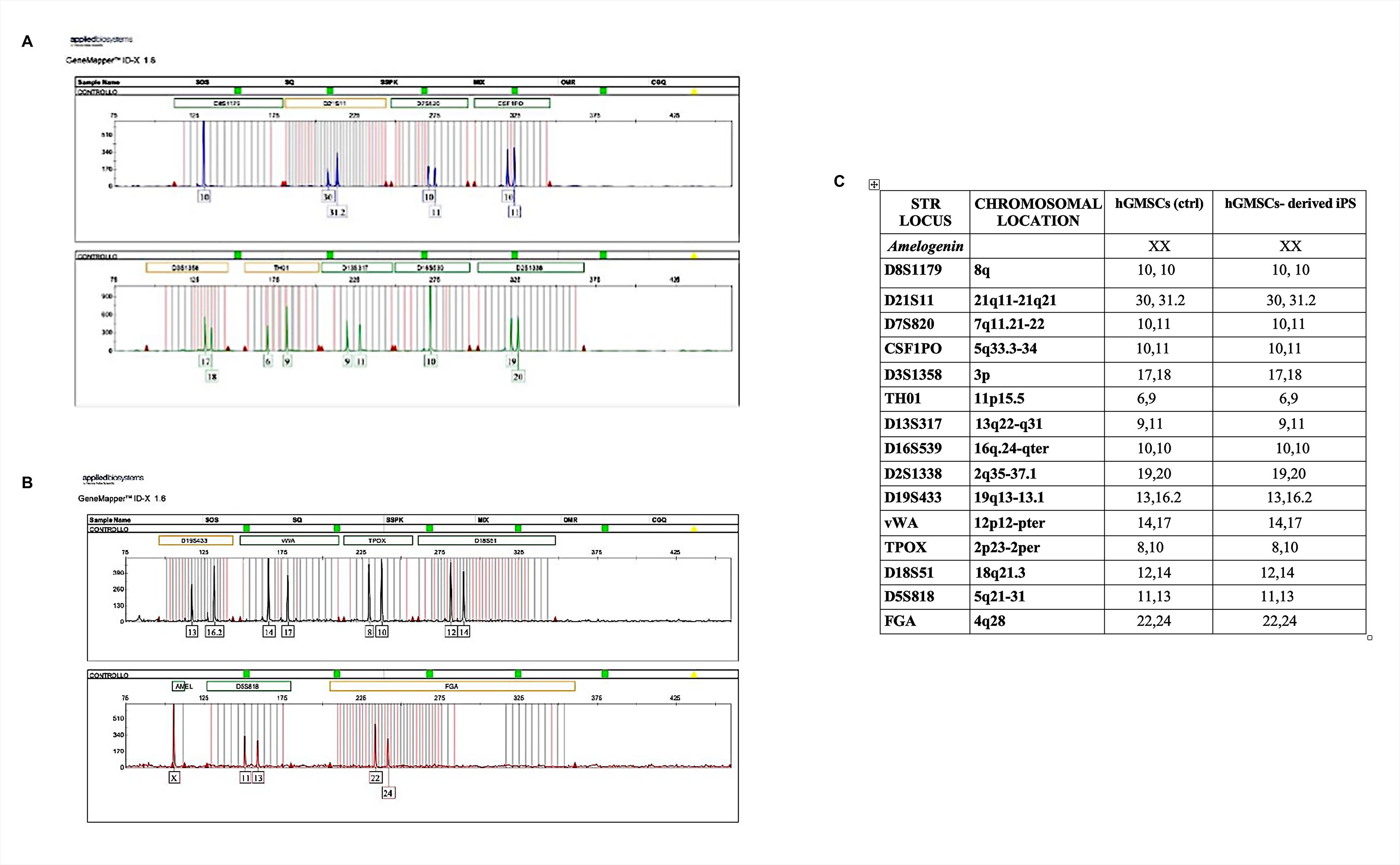

Supplement: Supplementary file 1 [file ijms-25-09169-s001.zip › Figure S3.tif]

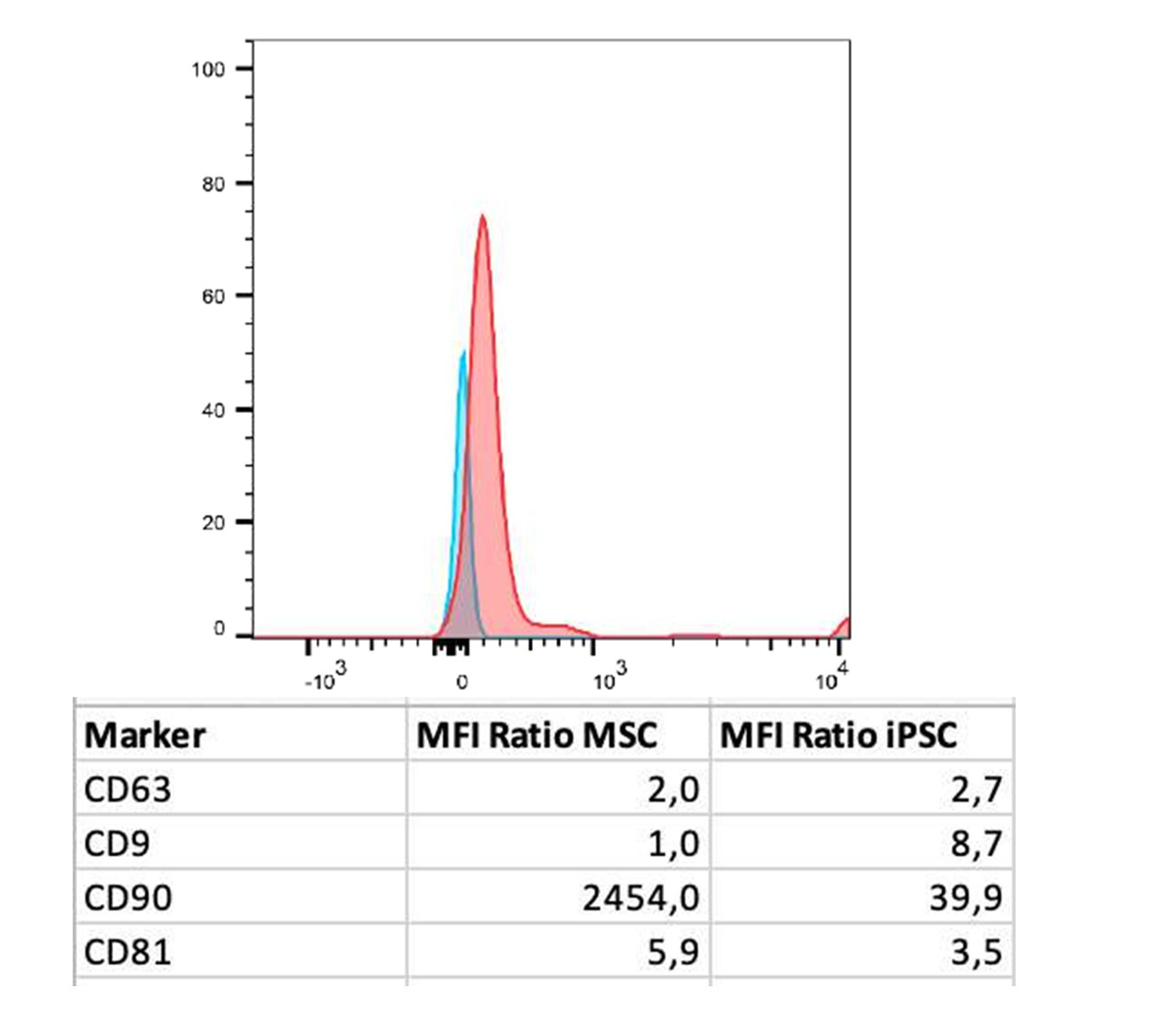

Supplement: Supplementary file 1 [file ijms-25-09169-s001.zip › Figure S4.tif]
